# Nonoperative treatment versus volar locking plate fixation for elderly patients with distal radial fracture: a systematic review and meta-analysis

**DOI:** 10.1186/s13018-020-01734-2

**Published:** 2020-07-14

**Authors:** Qiang Li, Chao Ke, Shuang Han, Xin Xu, Yu-Xuan Cong, Kun Shang, Ji-Dong Liang, Bin-Fei Zhang

**Affiliations:** 1 Department of Hand Surgery, Honghui Hospital, Xi’an Jiaotong University, No. 555 Youyi East Road, Beilin District, Xi’an, 710054 Shaanxi Province People’s Republic of China; 2 Department of Orthopedic Trauma, Honghui Hospital, Xi’an Jiaotong University, No. 555 Youyi East Road, Beilin District, Xi’an, 710054 Shaanxi Province People’s Republic of China

**Keywords:** Volar locking plate, Nonoperation, Distal radial fractures, Systematic review, Meta-analysis

## Abstract

**Background:**

This systematic review and meta-analysis assessed the role of nonoperative treatment and volar locking plate (VLP) fixation in elderly patients with distal radial fracture.

**Methods:**

The systematic literature review identified randomized controlled trials (RCTs) and observational studies using VLP and nonoperative treatment for distal radial fractures in the elderly. Two investigators independently extracted data and evaluated the quality of the studies. A meta-analysis was performed using RevMan version 5.3.

**Results:**

The five RCTs and six observational studies included 585 and 604 patients in the VLP and nonoperation groups, respectively. The quality of these 11 studies was moderate. Compared to nonoperation treatment, VLP did not improve the disabilities of the arm, shoulder and hand (DASH) score (weighted mean difference [WMD] = −1.67; 95% confidence interval [CI], −3.58–−0.24; *P* = 0.09), decrease complications (odds ratio = 1.05; 95% CI, 0.51–2.19; *P* = 0.89), or improve range of motion in flexion, extension, pronation, supination, and radial deviation. The VLP group had better grip strength (WMD = 10.52; 95% CI, 6.19–14.86; *P* < 0.0001) and radiographic assessment than the nonoperation group.

**Conclusions:**

Although insufficient, the study evidence shows that VLP does not improve DASH scores, complications, or range of motion, but it might provide better grip strength and radiographic assessment than nonoperation treatment.

## Introduction

Distal radial fractures are the most common fractures encountered in health care [1], accounting for about 17.5% of all fractures in 2000, especially among elderly people. Generally, the initial treatment is closed reduction and fixation with casting. However, if a good reduction cannot be achieved in the first trial or sustained in later trials, a surgical option can be considered to obtain better reduction and acceptable radiological parameters. Considering the increasing life expectancy of the elderly population, the appropriate treatment of these fractures is of growing importance.

Stable fractures can be treated with closed reduction and cast immobilization, with satisfactory outcomes in the early stage [2]. For unstable fractures, closed reduction cannot be maintained with external immobilization and additional fixation is suggested [3]. Since locking plate fixation (VLP) introduction, there has been a tendency to manage distal radial fracture in elderly people with VLP [4] and the rate of operative treatment in the elderly has increased gradually over the decades [5]. However, until recently, surgical treatment with a volar locking plate for unstable fractures among the elderly population has not been proven to be superior to nonoperative treatment [6–8]. Martinez-Mendez et al. reported significantly better function for patients treated with a VLP compared to that in nonoperative treatment [9]. Arora et al. observed no differences in function, pain, and disability scores in a randomized controlled trial [6]. Some authors have suggested that elderly patients with distal radial fractures should be managed nonoperatively because fracture reduction and anatomic alignment on radiographs are not correlated with better functional outcomes in these patients [10–12], or the correlation is not clearly proven [13]. Thus, which VLP treatment or cast application is better in the treatment of distal radial fractures in elderly patients remains controversial.

Therefore, we performed a systematic review and meta-analysis to assess the role of VLP and nonoperation on distal radial fracture function to provide clinical guidance.

## Methods

### Inclusion and exclusion criteria

The inclusion criteria were as follows: (1) randomized controlled trials (RCTs) or prospective or retrospective controlled studies, (2) participants aged above 50 years and with distal radial fractures, (3) patients treated surgically with VLP or nonoperation treatment with casting, (4) reported outcomes including wrist function, radiographic assessment, and complications in follow-up, (5) follow-up of at least 12 months.

The exclusion criteria were case series study without comparison groups and studies not reporting on the outcomes of interest.

### Literature search

We searched the MEDLINE, Embase, and Cochrane library databases using the keywords volar plate, palmar plating, operation, surgery, distal radial fracture, radius fracture, nonoperati*, conservative, plaster, and cast. The retrieval dates included the time from database creation to September 2019. There was no limitation in the process of searching.

### Outcome measures

The endpoints were disabilities of the arm, shoulder, and hand (DASH) score, grip strength, complications, range of motion, and radiographic assessment. The grip strength was presented as percentages of the uninjured side. The range of motion included flexion, extension, pronation, supination, radial deviation, and ulnar deviation. The radiographic assessment included radial height, radial inclination, ulnar variance, and volar tilt. The complications include reduction loss, revision, rupture of tendons, wound infection, nerve lesion, carpal tunnel syndrome, and complex regional pain syndrome.

### Data extraction and quality evaluation

We screened all titles of the retrieved articles and removed duplicates. After eliminating irrelevant articles, the summaries of the remaining articles were assessed to confirm the adequacy of the information, followed by reading the full texts. Two investigators resolved disagreements through discussion and unresolved disagreements were discussed with a third investigator. We assessed the RCTs using Jadad scoring [14] for the generation of allocation sequence, allocation concealment, blinding, withdrawals, and efficacy of randomization. The Newcastle-Ottawa scale (NOS) was used to assess the nonrandomized studies [15].

### Statistical methods

Odds ratios (ORs) and weighted mean differences (WMDs) were used for effect sizes, with 95% confidence intervals (CIs). The statistical methods included Mantel–Haenszel and inverse variance tests. We assessed heterogeneity with *I*^2^ statistics. During quantitative synthesis, a fixed-effects model was employed for low heterogeneity (*I*^2^ < 50%, *P* > 0.1). When heterogeneity was high (*I*^2^ > 50%, *P* < 0.1), we first explored the possible sources of heterogeneity or used a random-effects model. *P* < 0.05 was considered a statistically significant difference. RevMan 5.3 version (The Cochrane Collaboration, Copenhagen, Denmark) was used to perform the analyses.

## Results

### Including studies

**Fig. 1:**
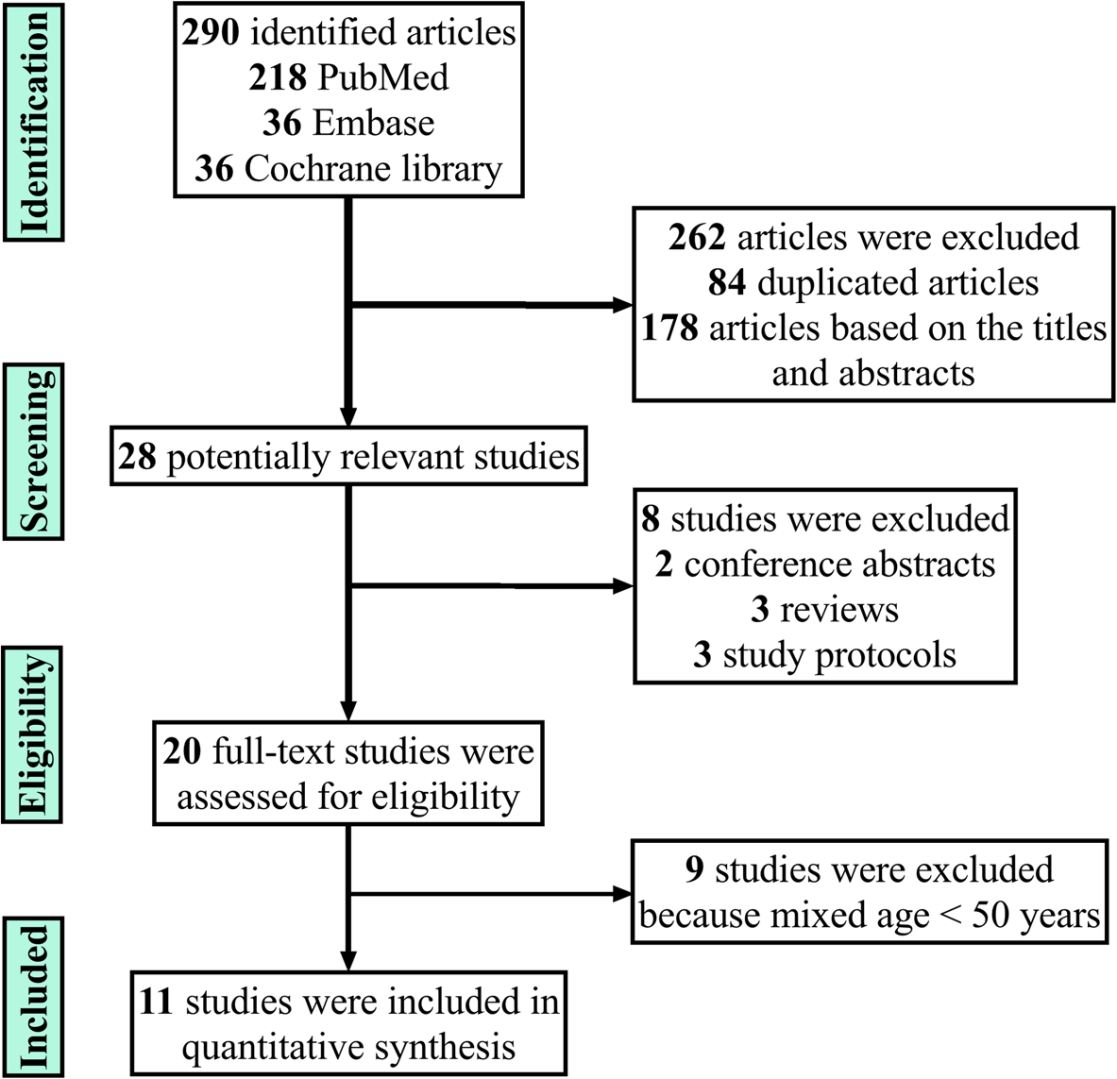
Flowchart of the studies included in the meta-analysis

Of 290 potentially eligible articles, most were excluded due to duplicates and lack of relevance. Finally, 11 studies [4, 6, 7, 9, 16–22], including five RCTs [6, 7, 9, 20, 21], five retrospective studies [4, 16–18, 22], and one prospective study [19], satisfied the inclusion criteria after screening and assessment. The studies were published from 2009 to 2019, most within the past 5 years. Figure 1 shows the flow of studies through the trial.

### Characteristics and quality evaluation of the included studies

Eleven studies with a total of 1189 patients were included. Of these, 585 were in the VLP group and 604 were in the nonoperation group. The sample sizes ranged from 67 to 258 patients. The 1005 women comprised 84.52% of all patients. The lowest age in all studies was more than 50 years and most were more than 65 years of age. The VLP was used in the operation group by ORIF. Casting and plaster splints were used in the nonoperation group. All studies evaluated wrist function and treatment complications. The follow-up times varied from 12 to 55 months, as shown in Table 1.

**Table 1:** Summary of the included studies

Study ID	Study design	OTA/AO classification	No. of patients		Female		Age		Treatment		Outcome measures	Follow-up
			VLP	Nonoperation	VLP	Nonoperation	VLP	Nonoperation	VLP	Nonoperation		
Arora 2009	Retrospective	AC	53	61	36	42	75.9 ± 4.8	80.9 ± 5.7	ORIF	Casting	Range of motion, grip strength, DASH, radiographic assessment, complications	55 m
Arora 2011	RCT	AC	36	37	28	27	75.9 (65-88)	77.4 (65-89)	ORIF	Casting	Range of motion, grip strength, DASH, radiographic assessment, complications	12 m
Bartl 2014	RCT	C	86	88	77	76	75.3 ± 6.7	74.4 ± 7.1	ORIF	Casting	Range of motion, DASH, radiographic assessment, complications	12 m
Chan 2014	Retrospective	AC	40	35	34	30	71.5 ± 5.2	75.8 ± 9.3	ORIF	Casting	Range of motion, grip strength, DASH, radiographic assessment	12 m
Egol 2010	Retrospective	ABC	44	46	36	40	73 ± 6.2	76 ± 7.0	ORIF	Casting	Range of motion, grip strength, DASH, radiographic assessment, complications	12 m
Hung 2015	Retrospective	ABC	26	31	21	24	65	64	ORIF	Casting	Range of motion, grip strength, DASH, radiographic assessment, complications	12 m
Lutz 2014	Prospective	ABC	129	129	118	118	74 ± 5	74 ± 5	ORIF	Casting	Radiographic assessment, complications	12 m
Martinez-Mendez 2018	RCT	C	50	47	39	37	67 ± 8	70 ± 7	ORIF	Casting	Range of motion, grip strength, DASH, radiographic assessment, complications	24 m
Saving 2019	RCT	AC	58	64	55	56	80 (70-90)	78 (70-98)	ORIF	Plaster splint	Range of motion, grip strength, DASH, radiographic assessment, complications	12 m
SIRNIÖ 2019	RCT	AC	38	42	37	39	62 (50-79)	64 (50-82)	ORIF	Casting	Range of motion, grip strength, DASH, radiographic assessment, complications	24 m
Zengin 2019	Retrospective	C	25	24	18	17	66.6 ± 7.4	68.9 ± 8.7	ORIF	Casting	Range of motion, grip strength, DASH, radiographic assessment, complications	12 m

The quality of the included studies was assessed according to the above-referenced criteria. Among the five RCTs, Arora 2011 [6] did not include detailed information about the generation of the randomization sequence; the remaining RCTs reported that the randomization sequence was generated by computers or random number tables. All RCTs performed allocation concealment in opaque envelopes. Arora 2011 [6] reported that the assessor of the radiographic outcomes was blinded, resulting in a low risk of bias. However, the other RCTs did not report blinding. All RCTs reported information about withdrawals and no patients were lost to follow-up. Thus, the quality of the five RCTs was moderate (Table 2). The NOS was used to assess the quality of the retrospective and prospective studies, as shown in Table 3. The total scores ranged from 4 to 7, corresponding to moderate quality. Overall, the quality of the included studies was moderate.

**Table 2:** Quality assessment of the included randomized controlled trials (RCTs)

Study ID	Generation of randomization Sequence	Allocation concealment	Blinding	Withdrawals	Total score
Arora 2011 [6]	1	2	1	1	4
Bartl 2014 [7]	2	2	0	1	5
Martinez-Mendez 2018 [9]	2	2	0	1	5
Saving 2019 [20]	2	2	0	1	5
SIRNIÖ 2019 [21]	2	2	0	1	5

**Table 3:** Quality assessment of the included retrospective and prospective studies

Study ID	Selection			Comparability	Outcome		Total score
	Representativeness of exposed cohort	Selection of non-exposed cohort	Ascertainment of exposure	Comparability of cohorts on the basis of the design or analysis	Assessment of outcome	Adequacy of follow up of cohorts	
Arora 2009 [16]	0	1	1	2	1	1	6
Chan 2014 [17]	0	1	1	2	1	1	6
Egol 2010 [18]	1	1	1	2	1	1	7
Hung 2015 [4]	1	1	1	2	1	1	7
Lutz 2014 [19]	0	1	1	1	0	1	4
Zengin 2019 [22]	0	1	1	2	1	1	6

## Primary endpoints

### DASH score and grip strength

**Fig. 2:**
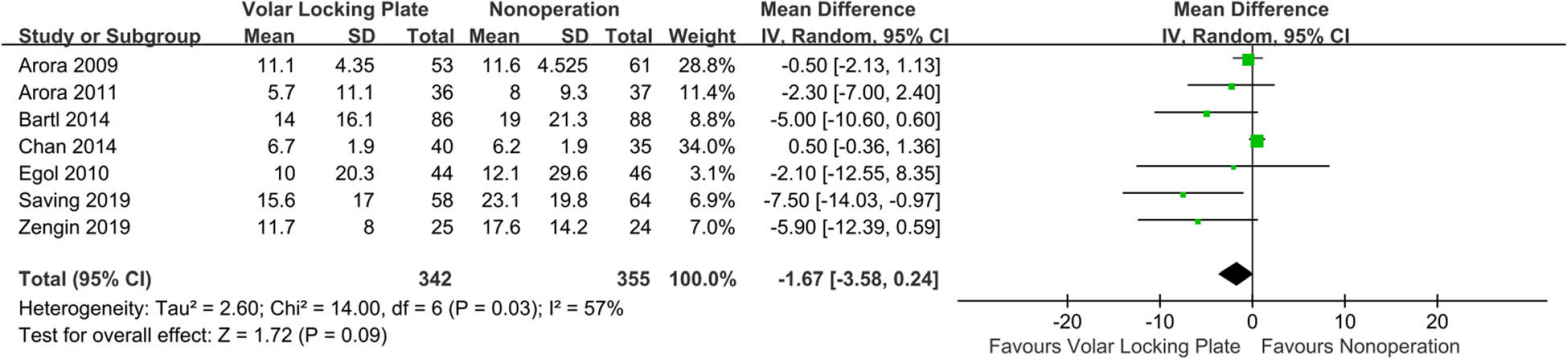
Forest plot comparing DASH scores between the VLP and nonoperation groups

Seven studies compared DASH scores between VLP and nonoperation groups [6, 7, 16–18, 20, 22]. As shown in Fig. 2, the *I*^2^ value for heterogeneity was 57% (*P* = 0.03). After excluding the possibility of clinical heterogeneity, a random-effects model was applied. The DASH score in the VLP group was comparable to that in the nonoperation group (WMD = −1.67; 95% CI, −3.58–−0.24; *P* = 0.09). When the aggregate results of these studies were divided into two subgroups according to the study design, the results from RCTs showed a lower DASH score in the VLP group than that in the nonoperation group (WMD = −4.37; 95% CI, −7.52–−1.21; *P* = 0.007). There were no significant differences in the results from retrospective studies (WMD = −0.20; 95% CI, −1.60–1.21; *P* = 0.78).

**Fig. 3:**
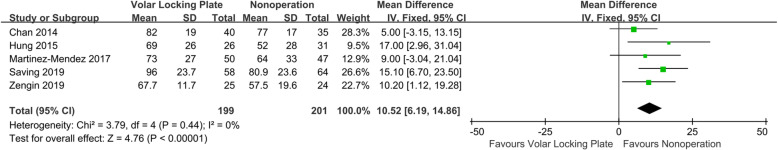
Forest plot comparing grip strength between the VLP and nonoperation groups

Five studies evaluated grip strength [4, 9, 17, 20, 22]. As shown in Fig. 3, since the *I*^2^ value for heterogeneity was 0% (*P* = 0.44), a fixed-effects model was used. The VLP group had significantly better grip strength than that in the nonoperation group (WMD = 10.52; 95% CI, 6.19–14.86; *P* < 0.0001). The results remained stable in the sensitivity analysis that excluded individual studies.

### Secondary endpoints

#### Complications

**Fig. 4:**
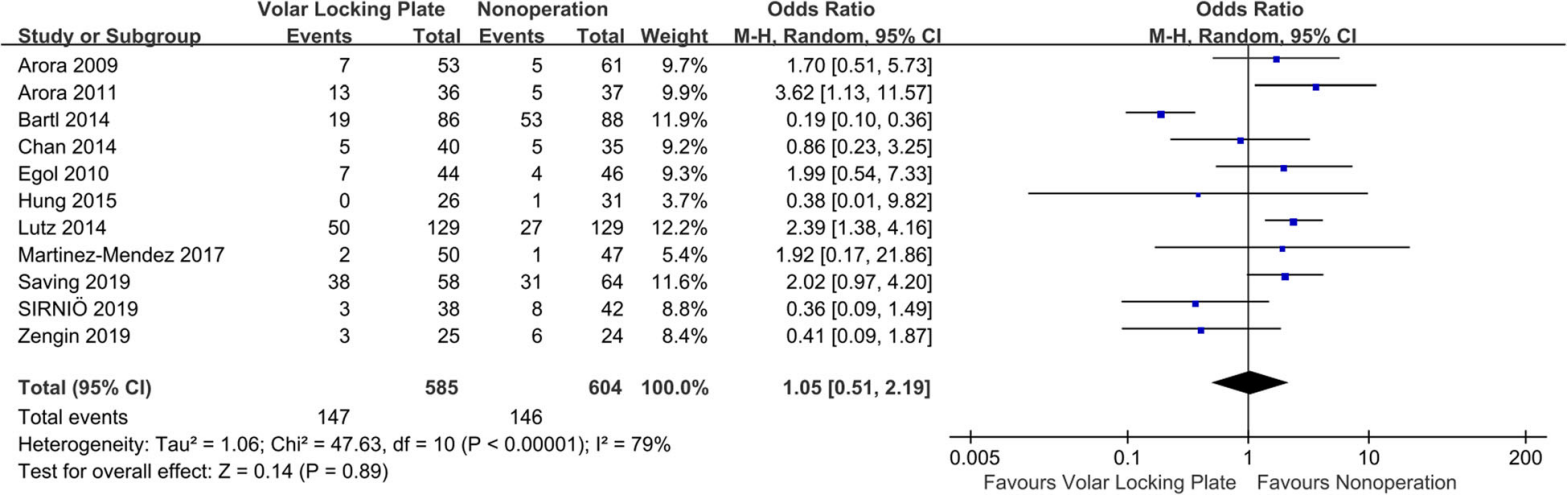
Forest plot comparing complications between the VLP and nonoperation groups

Eleven studies reported complications [4, 6, 7, 9, 16–22]. As shown in Fig. 4, the aggregate resulted in an *I*^2^ value for heterogeneity of 79% (*P* < 0.0001); thus, a random-effects model was used. No significant difference in the rate of complications (OR = 1.05; 95% CI, 0.51–2.19; *P* = 0.89) was observed between groups. The results remained stable in a sensitivity analysis that excluded individual studies. When the aggregate results of these studies were divided into two subgroups according to the study design, the RCTs showed no significant difference in complications between the VLP and nonoperation groups (OR = 0.94; 95% CI, 0.24–3.60; *P* = 0.92). There were also no significant differences in the results of the observational studies (OR = 1.47; 95% CI, 0.84–2.60; *P* = 0.18).

#### Range of motion

Most studies assessed the range of motion. As shown in Table 4, the aggregate results showed *I*^2^ values for heterogeneity in flexion, pronation, supination, radial deviation, and ulnar deviation of more than 50%; thus, the random-effects model was used. A significant difference between groups was observed only for ulnar deviation (WMD = 2.22; 95% CI, 0.19–4.26; *P* = 0.03), in which the ulnar deviation in the VLP group was higher than that in the nonoperation group. There were no significant differences in the field of flexion, extension, pronation, supination, and radial deviation.

**Table 4:** Range of motion and radiographic assessment of retrospective and prospective studies

Items	Number of studies	Mean difference [95% CI]	*P*	Heterogeneity	
				*I*^2^ (%)	*P*
Range of motion					
Flexion	7 [6, 7, 9, 16, 18, 20, 21]	−0.45 [−5.14, 4.24]	0.85	87	< 0.00001
Extension	7 [6, 7, 9, 16, 18, 20, 21]	−0.29 [−1.82, 1.23]	0.71	0	0.42
Pronation	7 [6, 7, 9, 16, 18, 20, 21]	1.03 [−1.08, 3.15]	0.34	71	0.02
Supination	7 [6, 7, 9, 16, 18, 20, 21]	1.42 [−1.37, 4.20]	0.32	78	0.0001
Radial deviation	6 [6, 7, 16, 18, 20, 21]	−0.21 [−1.37, 0.95]	0.72	26	0.24
Ulnar deviation	6 [6, 7, 16, 18, 20, 21]	2.22 [0.19, 4.26]	0.03	62	0.02
Radiographic assessment					
Radial height	4 [4, 9, 18, 22]	2.44 [1.22, 3.67]	< 0.00001	79	0.003
Radial inclination	11 [4, 6, 7, 9, 16-22]	3.81 [2.92, 4.70]	< 0.00001	63	0.002
Ulnar variance	10 [4, 6, 7, 9, 16-19, 21, 22]	−0.89 [−1.92, 0.13]	0.09	94	< 0.00001
Volar tilt	11 [4, 6, 7, 9, 16-22]	6.39 [0.18, 12.59]	0.04	97	< 0.00001

#### Radiographic assessment

Most studies performed radiographic assessment. As shown in Table 4, the aggregate results showed *I*^2^ values for heterogeneity in radial height, radial inclination, ulnar variance, and volar tilt of more than 50%; thus, the random-effects model was used. There were significant differences between the two groups in radial height (WMD = 2.44; 95% CI, 1.22–3.67; *P* < 0.00001), radial inclination (WMD = 3.81; 95% CI, 2.92–4.70; *P* < 0.00001), and volar tilt (WMD = 6.39; 95% CI, 0.18–12.59; *P* = 0.04). The radial height, radial inclination, and volar tilt in the VLP group were better than those in the nonoperation group. No significant differences in ulnar variance were observed (WMD = −0.89; 95% CI, -1.92–0.13; *P* = 0.09).

#### Publication bias

**Fig. 5:**
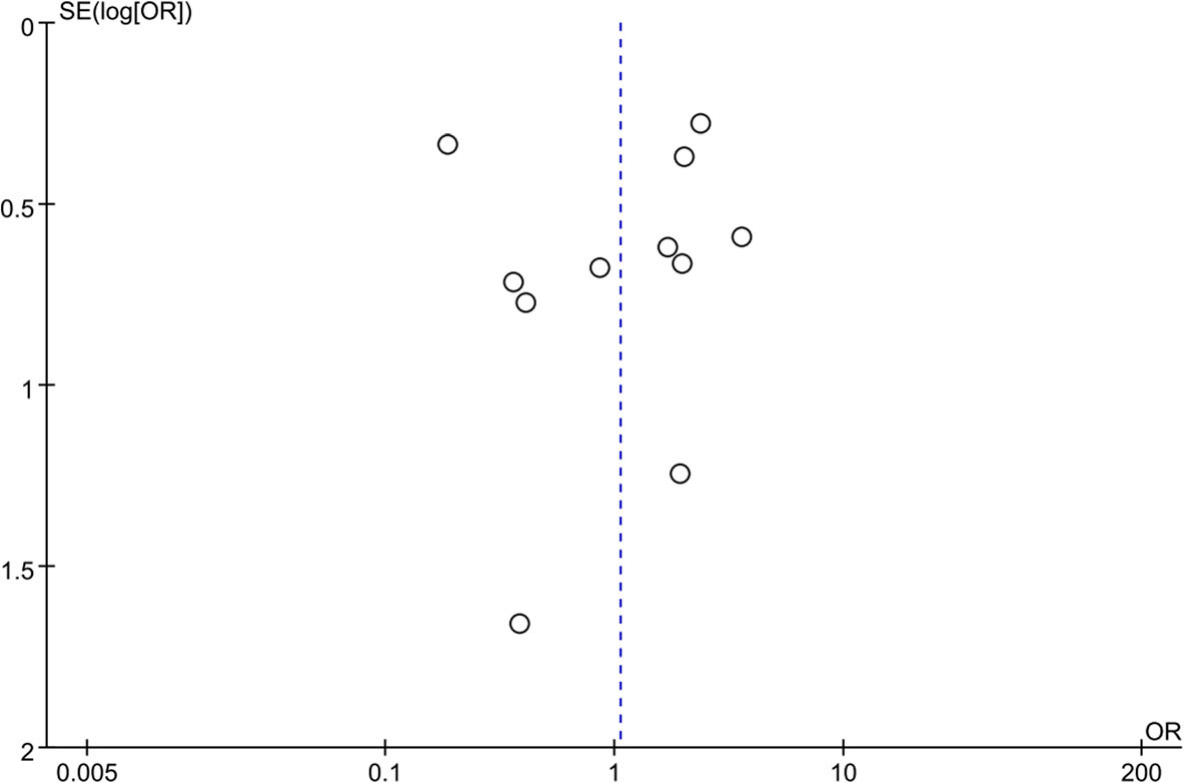
Funnel plot comparing complications between the VLP and nonoperation groups. The *y*-axis represents the standard error (SE) (log [OR]), while the *x*-axis represents the odds ratio (OR). The sloped lines represent the 95% CI boundaries and the circles indicate the 11 studies

Publication bias was assessed using complications for analysis. The symmetry shown in Fig. 5 suggested that publication bias was not likely.

## Discussion

Several recent studies have reported satisfactory functional results in elderly patients despite malunion [6, 16]. Some authors have suggested nonoperative management of elderly patients with distal radial fractures [10–12], and have demonstrated very good clinical outcomes in these patients [23]. Conversely, other authors have reported that articular reconstruction using VLP provides predictable results, especially in osteoporotic elderly patients [17]. In 2003, a Cochrane systematic review suggested that, despite the poorer radiological results, the functional outcome of nonoperative therapy did not differ from that of surgical management in patients over 60 years of age [24]. In their review of distal radius fracture in elderly patients, Ju et al. [8] reported similar functional outcomes and quality of life between operation and nonoperation. However, Handoll et al. [24] and Ju et al. [8] included external fixation, percutaneous pinning, ORIF, and scaffolding-bone graft/substitute in their operation groups; thus, the treatments varied. Studies comparing different surgical protocols [25, 26] concluded that ORIF with a plate might be the best surgical protocol for patients with distal radius fracture. Contrarily, other high-quality systematic review indicates there is no clear superiority between VLP and other common fixations [27]. The patient age in these systematic reviews varied widely and included elderly and non-elderly patients. The methods of reduction and fixation fracture in patients with osteoporosis differ from those in adults. The current literature on the treatment of distal radial fractures in the elderly remains controversial. Especially, in clinical practice, how should we give the weight to radiographic results and functional results? In a recent randomized study, Caruso et al. found radiological parameters outside the range conventionally considered acceptable do not preclude a satisfactory clinical outcome [28]. Thus, there is lack of the correlation between clinical and radiographic results in the elderly, and a strategy for elderly patients is required.

Thus, the present systematic review assessed the influence of VLP and nonoperation on distal radial fracture. The main findings were (a) no significant difference in DASH scores, complications, or range of motion and (b) better grip strength and radiographic assessment in the VLP group than those in the nonoperation group.

The primary outcomes in the present study were DASH score and grip strength. The DASH is an effective tool for assessing wrist functional disability in distal radial fractures [29, 30]. No differences in range of motion and complications were observed between the VLP and nonoperation treatment groups. Therefore, the functional results of the two methods do not differ significantly. The result is similar to that of another meta-analysis containing only elderly patients [8]. The grip strength of the surgical group was significantly higher than that of the nonoperation group. In terms of radiographic evaluation, VLP improved four parameters after ORIF of the distal radius fracture. However, in elderly patients with mainly functional requirements, the recovery of the appearance of the wrist and improved radiographic parameters may be less important than the function. Therefore, there was little difference in function between the two groups, but there were differences in grip strength. In elderly patients, if the expected quality of life is high or the injured wrist is on the advantage upper extremity, VLP treatment may achieve better results. If the patient is older, the expected quality of life is not high, or if the injury wrist is on the disadvantage upper extremity, conservative treatment should be considered.

The complications include reduction loss, revision, tendon rupture of, wound infection, nerve lesion, carpal tunnel syndrome, and complex regional pain syndrome. Carpal tunnel symptoms, reduction loss, and complex regional pain syndrome occurred more often in the nonoperation group [7]. Revision, tendon rupture, wound infection, and nerve lesions occurred more often in the VLP group [19].

Our meta-analysis has several limitations. First, the study included RCTs and observational studies. One study reported that observational studies may exaggerate the actual role of VLP [31]. Second, a slight statistical heterogeneity was observed among the included studies, which could have affected the results. Third, the quality of these studies was moderate. Thus, the results should be cautiously interpreted. A large-scale study is needed to identify the role of VLP and nonoperation. Fourth, the use of arthroscopy is increasingly popular in the last years and providing a better anatomical reduction [32], but VLP group in most of studies do not report the usage of arthroscopy. In addition, the issue of the difference between articular and extra-articular fractures is never addressed. This could affect the results and could be another inherent limitation of the study.

## Conclusions

Although insufficient, the evidence from this study showed that VLP might not improve the DASH score, complications, or range of motion. VLP might provide better grip strength and radiographic assessment compared to nonoperation.

## Data Availability

Data sharing is not applicable to this article as no datasets were generated or analyzed during the current study

## Abbreviations

VLP: Volar locking plate
RCT: Randomized controlled trials
DASH: Disabilities of the arm, shoulder, and hand
WMD: Weighted mean difference
CI: Confidence interval
NOS: Newcastle-Ottawa scale
OR: Odds ratios
